# Canadian Cancer Centre Response to COVID-19 Pandemic: A National and Provincial Response

**DOI:** 10.3390/curroncol28010026

**Published:** 2020-12-31

**Authors:** Rebekah Rittberg, Anmol Mann, Danielle Desautels, Craig C. Earle, Sri Navaratnam, Marshall Pitz

**Affiliations:** 1 Section of Hematology/Oncology, Department of Internal Medicine, Rady Faculty of Health Sciences, University of Manitoba, Winnipeg, MB R3A 1R9, Canada; rrittberg@cancercare.mb.ca (R.R.); ddesautels@cancercare.mb.ca (D.D.); 2 CancerCare Manitoba, Winnipeg, MB R3E 0V9, Canada; snavaratnam@cancercare.mb.ca; 3 Rady Faculty of Health Sciences, University of Manitoba, Winnipeg, MB R3E 3P5, Canada; manna34@myumanitoba.ca; 4 Sunnybrook Health Sciences Centre, Toronto, ON M4N 3M5, Canada; Craig.Earle@partnershipagainstcancer.ca

**Keywords:** coronavirus, COVID-19, health service research, healthcare delivery, operations, pandemic, oncology, public health practices, treatment, telemedicine

## Abstract

Background: COVID-19 has spread rapidly, requiring health delivery systems to undertake dramatic transformations. To evaluate these system changes, we undertook one of the first Canadian health delivery system reviews and the first Canadian cancer centre evaluation of pandemic system modifications. Methods: Questionnaires were distributed to the Canadian Association of Provincial Cancer Agencies (CAPCA) members in order to assess changes to cancer centre services and patient management. Documentation relating to COVID-19 from the CAPCA electronic space was accessed, and all publicly available cancer centre documentation related to COVID-19 was reviewed. Results: Seven provinces completed the questionnaire and had documentation available from the CAPCA electronic space. All screening programs across Canada were suspended. In most provinces surveyed, ≥50% of outpatient appointments were occurring virtually, with <25% using video platforms. Generally, the impact on diagnostic imaging and new patient referrals correlated with the impact of COVID-19. Most provinces had a reduction in operating room availability, with chemotherapy and radiation treatments continuing. Public health modification, including personal protective equipment and screening staff, varied across the country. Conclusion: Canadian cancer centres underwent a rapid and aggressive transformation of services in response to COVID-19, with many similarities and differences across provinces. In part, this response was facilitated by communication under a national association, which in Canada remains unique to cancer. This response may serve to inform changes in other jurisdictions or disease states now and in future waves of the pandemic, as well as a record of changes for future health services and patient outcome research.

## 1. Introduction

Severe acute respiratory syndrome coronavirus 2 (SARS-CoV-2) and its resulting illness, COVID-19, was first recognised in December 2019 in Wuhan, China. COVID-19 spread globally, with the first case identified in Canada on 15 January 2020 [1,2,3]. With variable presentations and severities of illness, COVID-19 can be an unpredictable and lethal virus [1,4,5]. In early phases of the pandemic, it became apparent that elderly patients and those with underlying health conditions were more commonly and severely affected [6,7]. Cancer patients may carry an additional risk if receiving immunomodulating or immunosuppressive treatment [8,9,10]. Additionally, cancer patients receiving treatment are exposed to healthcare workers that may be infected by COVID-19. These workers may be asymptomatic or mildly symptomatic, allowing for continued work while unknowingly being infective [11].

In response to the COVID-19 pandemic, cancer care delivery has undergone rapid transformation [12,13]. Although journals are ensuring rapid peer review and publication of articles pertaining to COVID-19, most explore presentation, treatment and outcome, while little has been published about system-level responses [14]. Understanding how healthcare systems have adapted, the impact on non-COVID-19 infected patients and implications for patients with cancer is integral to ensuring optimal patient care during the pandemic. Documenting these changes will also provide context for understanding health services and patient outcome research moving forward [15,16].

In March 2020, at the onset of Canada’s COVID-19 pandemic, Canadian cancer centres underwent a rapid and aggressive transformation in patient care delivery. These changes were undertaken at a provincial level with some national coordination. The Canadian Association of Provincial Cancer Agencies (CAPCA) is a Canadian interprovincial organisation comprised of senior members from each provincial cancer program, whose aim is to strengthen national cancer care delivery. CAPCA allows for regular communication between cancer centres, and its members meet on a weekly basis to discuss changes, successes and challenges from the onset of the pandemic. The national framework of CAPCA allows for a unique review of national trends not possible in other medical specialties. 

As we continue to adjust our practices to the ongoing COVID-19 pandemic, we must acknowledge and study the impact of these drastic measures. We undertook one of the first national health delivery system reviews and the first evaluating Canadian cancer centre responses to the COVID-19 pandemic. We sought to describe the changes that occurred as a basis for a broader discussion of health system responses to the pandemic in Canada and abroad, to understand the immediate challenges resulting from those changes and to facilitate the interpretation of future health services and patient outcome research in the context of changes made as a result of the pandemic.

## 2. Methods

With University of Manitoba Health Research Ethics Board approval, we distributed a questionnaire to CAPCA members. This research project has University of Manitoba Health Research Ethics Board approval. The ethical code number is HS23999 (H2020:275). Approval date is 15 May 2020 and expiry date is 15 May 2021. The questionnaires were answered by cancer centre Chief Executive Officers (CEOs), with input from delegates based on the required information. The questionnaire considered modifications taken by cancer centres across Canada resulting from the COVID-19 pandemic. Specific areas of interest included: cancer screening, cancer diagnostics, outpatient appointments, inpatient services, treatments (surgery, chemotherapy, and radiation), cancer centre operations, trainee management and research; see Supplemental Appendix 1. All questions had categorical answers and considered the extent to which normal centre operations had been affected as well as the methods employed to deal with the impact. Responses included both measured outcomes and estimates across multiple different cancer centres within a single province. 

The CAPCA electronic space, a private cloud used by members to share documents relating to the coordinated management of the cancer system, was accessed and all documentation relating to COVID-19 was reviewed. This was available for British Columbia (BC), Alberta (AB), Saskatchewan (SK), Manitoba (MB), Ontario (ON), Quebec (QC) and Nova Scotia (NS). Each Canadian cancer centre website was reviewed for public documentation pertaining to COVID-19. Authors (RR, AM and DD) reviewed the CAPCA electronic space and cancer centre websites. A descriptive analysis was completed to evaluate similarities and differences between provincial responses.

## 3. Results

Completed questionnaires were received from seven provinces: BC, AB, SK, MB, ON, QC and Newfoundland and Labrador (NL); see Table 1. Canadian provincial cancer centres publicly provided varying amounts of COVID-19 pandemic response information online. This public information was reviewed, as well as the CAPCA electronic space; see Supplemental Table S1. The absence of a specific provincial response only indicates that the information is not available. 

### 3.1. Screening Programs

Most cancer centres across Canada suspended cancer screening programs for breast, cervical and colon cancer in mid-March 2020. The intent of the closure was to support physical distancing measures and to aid in the redeployment of essential healthcare workers. Patients who had recently undergone screening, prior to program closures, were triaged based on acuity. Patients who had recently undergone screening with results highly suspicious for malignancy were evaluated for a biopsy or additional investigations. Patients with an abnormal screening result not highly suspicious for malignancy had follow-up appointments delayed. Cancer screening programs started to reopen in June 2020. 

### 3.2. Outpatient Appointments

A decrease in new patient referrals occurred across Canada, with a >20% decrease in SK, ON and QC. MB experienced a 10–20% reduction in referrals and noted a decrease in incoming pathology reports, from approximately 1000 during the week of 16 March 2020, and dropping to as low as 600 in the week of 6 April 202020. 

Appointments moved toward the telemedicine/virtual space across the country to minimise in person appointments and maximise efficiency in a resource strained environment. Of the seven provinces that responded to the questionnaire, six indicated that ≥50% of outpatient appointments are occurring virtually, with the initial patient consultation being most likely to occur in person. Provinces restricted family members/friends from accompanying patients to appointments. Exceptions were permitted, including (but not limited to) minors being assessed, patients with language barriers or patients with physical or cognitive impairments. 

On treatment appointments continued and at least 50% of appointments used virtual tools. Changes were relatively homogenous across Canada. Follow-up visits for patients not on active treatment were deferred at the discretion of the treating physician and utilised virtual tools 50–75% of the time. Video conferencing accounted for <25% of virtual appointments and platforms included Zoom, Pexip, Microsoft Teams, Reacts and Jabber. Patients were notified of upcoming appointments by letter sent by Canada Post or telephone, with only ON using a patient portal. 

### 3.3. Treatment

Cancer treatments were affected across all provinces. Internal strategies were developed and adopted by disciplines and disease site groups to provide guidance for providers. Most provinces saw a reduction in operating room availability, and elective surgeries were postponed. Oncologic surgeries were delayed at the discretion of treating physicians. Preference was given to neoadjuvant strategies, including approaches such as neoadjuvant endocrine therapy for hormone receptor positive breast cancer. In some provinces, surgical wait times decreased due to the reduction in cancer screening, diagnostic imaging and referrals. 

Administration of chemotherapy and radiation faced minimal or no changes. However, provinces prepared prioritisation guidelines for each disease site to prepare for a possible worsening of the pandemic. Hypofractionated and short course radiation treatments were recommended where possible, to reduce resource consumption and minimise patient exposure [17]. Similarly, the longest possible chemotherapy cycle length and protocols with the fewest number of appointments were considered. QC also recommended transitioning to longer cycles for immunotherapy, such as administration of nivolumab every 4 weeks as opposed to every 2 weeks. SK, ON and QC offered COVID-19 testing to asymptomatic patients on treatment. Several provinces indicated that supportive therapies such as adjuvant bisphosphonates for breast cancer should be deferred and monthly bisphosphonates for metastatic disease be converted to every 3 months. Multiple provinces suggested adding granulocyte colony stimulating factor as primary prophylaxis to reduce emergency room visits for febrile neutropenia. 

Patient cards were issued in SK and NS to ensure that patients on chemotherapy were easily identified in Emergency Departments. The intent was to serve as a reminder, in the era of COVID-19, that a fever in a patient receiving chemotherapy is febrile neutropenia until proven otherwise. 

### 3.4. COVID-19 Positive Patients

Cancer patients who tested positive for COVID-19 were restricted from entrance to cancer centres during the infective period, unless extenuating circumstances were present. Inpatient oncology patients with suspected or confirmed COVID-19 were admitted to a separate unit.

BC developed a test-based strategy for the resumption of cancer treatment subsequent to COVID-19 infection. Treatment may be resumed when all of the following criteria are met: resolution of fever (without use of antipyretics), resolution of all symptoms, two negative nasopharyngeal swabs (at least 24 h apart) and discussion with the Medical Heath Officer at Public Health. Similarly, AB recommended the resumption of cancer treatment after resolution of symptoms, at least 14 days from onset of symptoms and with two negative nasopharyngeal swabs (7 days apart). Delay in initiation of chemotherapy of >3 months was considered reasonable in low-risk patients, up to 3 months for intermediate-risk patients and no delay for high-risk patients. 

### 3.5. Investigations (Diagnostic Imaging and Phlebotomy)

Elective imaging was postponed to various degrees across Canada. BC and ON published guidelines on the prioritisation of imaging based on disease type and stage to be used if resources became limited. Investigations for cancer screening, low-risk post-treatment surveillance and treatment planning for slow growing tumors was considered low priority. Impact on diagnostic imaging varied across the country, with MB and SK having a <25% reduction in volume of computed tomography (CT), magnetic resonance imaging (MRI) and ultrasound, while QC and NL experienced a 50–75% reduction. 

Some provinces experienced changes to phlebotomy services, including reduced blood draws for stable indications and the redirection of patients to community laboratories. NS launched an in-home phlebotomy service for patients who were unable to leave their home due to mobility issues, chronic disease or isolation for COVID-19. 

### 3.6. Cancer Centre Operations

Across Canada, healthcare professionals required COVID-19 daily symptom screening before entering cancer centres. Variation was seen in screening protocols for staff, with AB using a daily self-completed questionnaire, while MB screened employees at the cancer centre entrance with questions and temperature monitoring. PPE practices for low-risk asymptomatic patient encounters also varied. Masks were required in all provinces, with the addition of eye protection in BC, MB and QC. The use of a gown/lab coat was only required in QC. 

Patients entering a cancer centre were screened for COVID-19 symptoms, travel history and COVID-19 exposure. Screening occurred either at the entrance, over the phone the day before or both.

Meetings between healthcare professionals took place using many platforms, including Zoom, Pexip, Microsoft Teams and Reacts. Some cancer centre employees, including physicians, nurses, administrators and allied health workers, were able to work from home. This varied across Canada, with <20% of institutional staff for BC and NL and 20–50% in AB, SK, MB and ON working from home. 

### 3.7. Advance Care Plan (ACP)

Many provinces provided additional direction to physicians to emphasise the importance of Health Care Directives and end of life discussions in oncology clinics to reduce downstream health system, patient and provider burden. BC highlighted the importance of an early goals of care conversation, with particular exploration of patient wishes around end of life due to the added risk of COVID-19. ON acknowledged that COVID-19 may result in an increased burden on Palliative Care resources and that other frontline workers will be required to help manage these patient needs. 

### 3.8. Academics

Clinical trials were affected to various degrees across the country. Clinical trials remained open in most circumstances, but patient accrual was paused provincially in MB, QC and NL, while some local sites were also paused in other provinces. Non-clinical trial research was entirely suspended in surveyed provinces, with the exception of BC, experiencing a 30–50% reduction. Data capture occurred in all cancer centres evaluating the volume of new patient referrals, follow-up appointments (including psychosocial oncology appointments) and treatment administered, allowing for this research to occur. BC and QC experienced no modifications to trainee management, with the remaining provinces reporting a reduced exposure to inpatient and outpatient oncology.

## 4. Discussion

Canada has a publicly funded universal healthcare system with prioritisation based on medical need and not the recipient’s ability to pay. Consequently, our system generally has longer wait times than a private healthcare system, highlighting the importance of efficiency [18]. Importantly, this also facilitates the coordination of the health system at the regional, provincial and national level. Cancer is a unique disease entity in this regard, and each Canadian province has an overarching organisational and administrative structure governing the care of patients with cancer based on provincial administration and funding of the health system. The fact that the provincial cancer agencies can respond in a similar manner is a result of CAPCA, and similar national associations should be encouraged for non-oncologic diseases. Collecting and reporting on positive and negative healthcare system changes due to the COVID-19 pandemic is critical in order to guide future health system policy, permit optimal planning for future waves of the pandemic and determine which positive changes might be sustainable in the long term. This evaluation of Canadian cancer centre modifications serves as a foundation for future research looking at cancer patient-related outcomes and interpretation of health system changes during the COVID-19 pandemic. 

Across Canada, as the pandemic developed, an immediate goal was to decrease traffic in cancer centres in order to limit exposure and protect both patients and cancer centre employees from COVID-19. This was accomplished through multiple interventions. First, most medical appointments were converted to virtual, with certain necessary exceptions. Second, family members/friends were prevented from accompanying patients to appointments. Third, staff were encouraged to work remotely where possible. Fourth, non-urgent diagnostic imaging and bloodwork was reduced.

These physical distancing measures continue to be extremely important as we increasingly recognise the risk of asymptomatic COVID-19 carrying healthcare workers to patients. As the pandemic has progressed, we have learned the importance of not only screening patients but also healthcare workers [11,19]. As an additional method to reduce the risk of asymptomatic spread from healthcare workers to vulnerable patients, some healthcare institutions completed periodic rapid antigen screening of healthcare workers, which successfully identifies asymptomatic carriers. Although not completed at any Canadian cancer centres, periodic rapid antigen screening may be a future method employed if the risk of asymptomatic COVID-19 carrying healthcare workers is felt to be high [20].

A 10–20% decrease in referrals was observed in most provinces, with SK, ON and QC observing a >20% reduction. This was likely due to a reduction in access to primary healthcare providers, screening programs and/or diagnostic services, leading to a reduction in new cancer diagnoses, in addition to patient hesitation to seek medical care out of fear of infection. There was a variable reduction in diagnostic imaging, generally mirroring the severity of COVID-19 pandemic. One of the first measures taken by cancer programs was the temporary closure of cancer screening programs. This was generally perceived as a low-risk measure; however, the consequences of screening program closures and resulting backlog will not be fully understood for years. It is unclear to what degree later stage/more advanced malignancies will be diagnosed due to the interruption in cancer screening and possible delay in seeking medical attention [21]. Having these system changes well-documented is critical to provide future context for population-based outcomes.

Telemedicine has been employed with success in past pandemics, including SARS-CoV (severe acute respiratory syndrome-associated coronavirus) and Ebola [22,23]. It has also been validated in heart failure and diabetic patients [24]. While not commonly used prior to the pandemic, in part due to the absence of provincial physician billing codes and privacy concerns, telemedicine has quickly become integral to cancer care. At the height of the COVID-19 pandemic, virtual appointments (video or telephone) made up 50–75% of oncology clinic schedules. While the use of video also allows for a limited examination, video platforms made up <25% of virtual appointments. Telemedicine has limitations, including technical issues, accessibility, inability to complete a full physical examination and privacy considerations. However, there are added benefits to patients, including reduced time commitment and financial strain of transportation and parking, thereby potentially reducing some inequity in access to services. Interestingly, no Canadian cancer centre reported communicating with patients using an electronic patient portal. 

We observed many similar trends across provinces, and although these decisions were made independently, they demonstrate a degree of homogeneity and coordination in Canadian practice. The rate of conversion of outpatient appointments to occur virtually was similar across Canada, with new patient appointments occurring most commonly in person, with virtual means used more frequently for on treatment and follow-up appointments. Generally, radiation and systemic therapy treatments required less modification and continued largely unaffected in most provinces. Surgery was impacted to a greater degree due to surgical resources directly competing with pandemic resources (including ventilators, large volumes of PPE and hospital rooms for patient recovery). Interestingly, more variation between provincial practices pertained to public health practices and not oncology-specific practices. There was no consistency between provinces in PPE use for asymptomatic patients or staff and patient screening. 

Early in the COVID-19 pandemic, it became apparent that cancer patients likely had a higher incidence of infection and higher risk of mortality compared to patients without a cancer diagnosis [9,10]. This changed the risk–benefit considerations of treatment and reinforced the importance of discussing goals of care and ensuring a realistic understanding of the severity of medical illnesses [25]. Globally, resources were stretched, resulting in restrictions in intensive care admission in some countries based on age and comorbidity. With the added threat of COVID-19, many provinces recommended that physicians undertake early goals of care conversations, with a particular focus on patient wishes around end of life. 

Transparency in cancer centre modifications and potential resource restrictions was extremely variable between provinces; see Supplemental Table S1. The larger cancer programs, including BC, AB and ON, provided some of the most comprehensive publicly available clinical management guidelines. Other provinces only provided documentation immediately applicable to patients and their care, including appointment changes, management of new symptoms or availability of additional mental health resources. 

We were limited by the extent of information available online and through the CAPCA electronic database. Not all provinces completed questionnaires, but we received robust responses from seven provinces, including the largest provinces. For larger provinces, the responses may be overall estimates for the entire province and may not be specifically representative of an individual cancer centre. Questionnaires were completed by cancer centre CEOs with input from delegates, resulting in the most complete information. However, respondents may have been limited by the data available to them. Importantly, we have not captured patient or provider experiences. We also lack complete information regarding the extent of the impact on clinical trials, which is a critical component of oncology care. Additionally, although we know that follow-up appointments, diagnostic imaging and oncologic surgeries were deferred, and treatment approaches were modified, we do not have a true measure of the proportion of patients affected or differences between provinces. 

At the time of submission, we enter the seventh month of the COVID-19 pandemic and provinces are in various stages of reopening. However, we are also beginning to recognise the chronicity of this pandemic. Which modifications are sustainable in the long term is not yet clear; however, many may be required until a vaccine is developed or herd immunity is established [16]. Many measures taken, including closure of cancer screening programs and delay of thousands of surgeries, were extreme and the true repercussions of these decisions may not be known for many years. 

## 5. Conclusions

Due to the COVID-19 pandemic, Canadian cancer centres undertook drastic changes to protect their vulnerable population. This transformation was in part coordinated by regular communication between provincial cancer agencies through CAPCA and highlights the importance of provincial, regional and national organisations in coordinating a response of this magnitude. To date, in Canada, we have minimal objective health system data documenting changes undertaken resulting from the pandemic and this research serves as an important foundation. 

We found many similarities between provinces, including closure of screening programs, transition to telemedicine and reductions in diagnostic imaging and new referrals. Differences also existed including public health practices, platforms used for virtual appointments and transparency. Many of these changes will be in place for months to come, and it is highly likely that we will require many years to fully understand the repercussions of the extreme measures used in response to the first wave of COVID-19.

## Figures and Tables

**Table 1:** Provincial responses to COVID-19 pandemic questionnaire.

	British Columbia	Alberta	Saskatchewan	Manitoba	Ontario	Quebec	Newfoundland
**Screening Programs:**							
Which screening programs were cut back or closed during the height of the COVID-19 Pandemic?	Breast, Colorectal and Cervical	Breast, Colorectal and Cervical	Breast, Colorectal and Cervical	Breast, Colorectal and Cervical	Breast, Colorectal and Cervical	Breast and Colorectal	Breast and Cervical
Which screening programs are to be re-opened in the next 4 weeks?	Reopening has begun	Reopening has begun	Reopening has begun	Reopening has begun	Reopening has begun	Reopening has begun	Reopening will soon begin
**Diagnostics:**							
What degree of reduction have the following diagnostic services experienced?							
CT	↓↓	↓↓	↓	↓	↓↓	↓↓↓	↓↓↓
Ultrasound	↓↓	↓↓	↓	↓	↓↓	↓↓↓	↓↓↓
MRI	↓↓	↓↓	↓	↓	↓↓	↓↓↓	↓↓↓
Nuclear Medicine	↓↓	↓↓↓	↓↓↓	↓	↓↓	↓↓↓	↓↓↓
Echocardiography	↓↓	↓↓	None	↓	↓↓	↓↓↓	↓↓↓
CT/Ultrasound guided biopsy	↓↓	↓	Variable depending on disease site	↓↓	↓↓	↓↓↓	↓↓↓
Has the reduction been for a specific indication?	N/A	Routine outpatient imaging studies and procedures	Follow-up	Unknown	Follow-up	N/A	Staging
How long do you expect diagnostic restrictions to be in place?	<4 weeks	>8 weeks	<4 weeks	N/A	4–8 weeks	>8 weeks	4–8 weeks
Have there been any changes in location that blood work is being completed?	No change	Shift away from cancer centre	Shift away from cancer centre	Shift away from cancer centre	Shift away from cancer centre	Varied by centre	No change
**Outpatient Appointments:**							
Has there been a decrease in new referrals to your cancer centre?	10-20% decrease	<10% (regional cancer centres), 10–20% (tertiary cancer centres)	>20% decrease	10–20% decrease	>20% decrease	>20% decrease	10–20% decrease
Are you expecting a surge of new referrals once you re-open?	<10% increase	10–20% increase	>20% increase	10–20% increase	10–20% increase	Yes, but cannot provide estimate	10–20% decrease
Are you screening/testing patients prior to entering the cancer centre?	Screening questions	Screening questions and temperature	Screening questions and temperature	Screening questions	Screening questions and temperature	Screening questions and temperature	Screening questions and temperature
Have you asked patients to come alone?	Yes (with exceptions)	Yes (with exceptions)	Yes (with exceptions)	Yes (with exceptions)	Yes	Yes (with exceptions)	Yes
What percentage of outpatient appointments are occurring virtually (video or telephone)?	↓↓↓	↓↓ (CCI, CACC), ↓↓↓ (TBCC, JACC)	↓↓↓↓	↓↓↓	↓↓↓	↓↓↓↓	↓↓↓↓
New patient appointments	↓↓	↓	↓↓	↓↓↓	↓	↓	↓↓
On treatment appointments	↓↓↓	↓↓ (CCI, CACC), ↓↓↓ (TBCC, JACC)	50%	↓↓↓	↓↓	Unknown	↓↓↓
Follow up appointments	↓↓↓	↓↓ (CCI, CACC), ↓↓↓ (TBCC, JACC)	↓↓↓↓	↓↓↓	↓↓↓	↓↓↓↓	↓↓↓↓
For virtual appointments, what percentage are delivered by video conferencing?	↓	↓	↓	Unknown	↓	↓	↓
For video virtual appointments, which platform is being used?	Zoom	Zoom	Pexip	Microsoft Teams	Zoom	Zoom, Reacts	Jabber
How are you communicating with patients about upcoming appointments?	Canada post, phone	Canada post, phone	Phone	Canada post, phone	Canada post, phone, patient portal	Phone	Canada post, phone
**Inpatient Wards/Consult Service:**							
Have admissions to inpatient units decreased?	<10% decrease	10–20% decrease	<10% decrease	>20% decrease	10–20% decrease	Unknown	>20% decreased
Has staffing of inpatient units changed?	No change	No change	No change	No change	No change	Fewer staff due to infection/quarantine	Fewer staff due to infection/quarantine, more staff due to inpatient volume
Are patients being screened or tested for COVID-19 prior to admission?	Screening questions and temperature	Screening questions	Screening questions and temperature	Nasopharyngeal swab	Screening questions, temperature and nasopharyngeal swabs	Screening questions, temperature and nasopharyngeal swabs	Screening questions and temperature
How are you handling suspect or confirmed COVID-19 patients on your oncology wards?	Separate unit	Separate unit	Separate unit	Separate unit and negative pressure rooms	Separate unit	Separate unit and positive patients sharing rooms	Separate unit
**Surgery:**							
Have there been any delays or deferral of cancer surgeries?	None	<4 weeks	<4 weeks	Minimally impacted, unknown duration	6–8 weeks	Surgery volume was reduced/unclear duration	>8 weeks (urgent and emergent cases proceeded)
Have cancer surgeries been prioritized?	No change	Prioritization within disease sites	No change	Prioritization within disease sites	Prioritization within disease sites	Prioritization within disease sites, disease type and to maximize surgical volume	Prioritization within disease sites
What will limit increases in surgical volume once you begin to re-open?	No change	COVID-19 precaution procedures	Access to PPE, COVID-19 precautions procedures, non-cancer surgery prioritization, staffing	No change due to minimal impact	COVID-19 precautions procedures, staffing	COVID-19 precautions procedures, non-cancer surgery prioritization, Staffing	Access to PPE, COVID-19 precautions procedures, non-cancer surgery prioritization
**Radiation:**							
Have there been any changes to the delivery of radiation therapy?	No change	No change	Changes to dose or fractionation schedule	Changes to dose or fractionation schedule	Changes to dose or fractionation schedule	Changes to dose or fractionation, prioritization within disease site and clinical indication	No change
How are you planning for increased volume once restrictions are eased?	No change	No change	Increased clinic hours	No change	No change	Increase in hours, use modified dose schedule, prioritization to specified disease sites and clinical indications	No change
**Systemic Therapy:**							
Have there been any changes to the administration of chemotherapy or supportive therapy?	No change	Varied depending on site: may have had changes to schedule, delayed where possible or no change	Changes to schedule (frequency, dose, density)	No change	Changes to schedule (frequency, dose, density)	Changes to schedule (frequency), prioritization within disease sites, disease type and clinical	Generally no change. Few cases had change to schedule (frequency, dose, density)
What additional systemic therapy considerations have been used?	No change	No change	Favour neoadjuvant and oral therapy, stopping maintenance therapy and G-CSF use	Favour oral therapy and G-CSF use	Favour oral therapy	Favour oral therapy and G-CSF	No change
Have you had to adjust the drug approval process/policy to allow more flexibility in choosing regimens?	No change	No change	No change	No change	Drug access liberalized for pandemic	Disease site-specific liberalization access granted	No change
Have you modified the use of satellite chemotherapy sites?	No change	No change	No change	No change	No change	No change	No change
What changes have occurred in the treatment room?	No change	Distancing patients, no escorts	Distancing patients, no escorts	Distancing patients, no escorts	Distancing patients, no escorts	Distancing of patients, no escorts and modified hours	Distancing patients
Are you testing patients prior to systemic therapy?	No	No	Offered, not required if asymptomatic	No	Yes, prior to cycle 1	Offered, not required if asymptomatic	No
Will you be testing patients prior to systemic therapy?	No	No	Offered, not required if asymptomatic	No	Yes, prior to cycle 1	Offered, not required if asymptomatic	No
Have you experienced any medication shortages?	No	Regional Cancer Centres experienced shortages with supportive care	No	No	No	No	No
**Advanced Care Planning (ACP):**							
Has there been direction to physicians to emphasize ACP or end of life care discussions in preparation for changes to access to ICU care?	No	Yes	Yes	No	Yes	Yes	Yes
Have you used video appointments for psychosocial oncology care?	50-75%	<25%	25-50%	>75%	25-50%	Unknown	None
Has the volume of psychosocial visits increased during this time?	No change	Decreased	<20% increase	Unknown	<20% increase	Unknown	No change
**Cancer Centre Operations:**							
What platforms are being used for case conferences?	Zoom	Zoom	Hybrid in person and video, Pexip, Webex	Microsoft Teams	Zoom	Zoom, Reacts	Microsoft Teams
Once restrictions ease, what services do you expect to continue long term?	Telephone visits, video visits, electronic communications with patients, video case conferences	Telephone visits, video visits, video case conferences	Telephone visits, video visits, electronic communications with patients, video case conferences	Telephone visits, video visits, video case conferences	Telephone visits, video visits, electronic communications with patients, video case conferences	Telephone visits, video visits, video case conferences	Telephone visits, video visits, video case conferences
Are some staff members working from home?	<20% Physicians, nurses, health records, administration, allied health	20–50% Physicians, administration	20–50% Physicians, allied health	20–50% Physicians, health records, allied health	20–50% Physicians, administration	Unknown percent Physicians, nurses, health records	<20% Physicians, nurses, health records, administration, allied health
What is the biggest barrier to working from home?	Paper/in-person workflow, lack of remote access, lack of devices	Paper/in-person workflow, lack of remote access	Paper/in-person workflow	None	Paper/in-person workflow	Paper/in-person workflow, lack of devices	Initially lack of devise, but this was resolved
Are patients or staff required to wear PPE?	All staff (masks)	All staff and patients (masks)	All staff and patients (masks)	All staff (surgical masks), patients may wear cloth masks	All staff and patients (masks)	All staff and patients (masks)	All staff and patients (masks)
What PPE are you using during a routine clinical encounter?	Eye protection and surgical/procedure mask	Surgical/procedure mask	Surgical/procedure mask	Eye protection and surgical/procedure mask	Surgical/procedure mask	Eye protection, surgical/procedure mask, gown and gloves	Surgical/procedure mask
Have there been concerns relating to shortages of PPE?	Yes, staff limited to one PPE set per day	Yes, using what is available	No	No, but cautious with usage (1 set available per day)	No	Yes, staff limited to one set per day and limiting trainees to preserve PPE	Yes, limited to one PPE set per day, limited number of providers seeing a patient and limit trainees to preserve PPE
**Research:**							
Are clinical trials currently open at your cancer centre?	Yes, all trials are in phase to be open or are open	Majority are open and only a small number of sponsor-driven trials were suspended	Yes, all trials are open	Yes, but not open to recruitment	Yes, but not open to recruitment	Yes, halted new patients on trials and new trials for adults, open for children	Yes, but not open to recruitment
Has non-clinical trial research continued?	Yes. Non-clinical research at 30–50% in person capacity	All research suspended	All research suspended	All research suspended	Clinical research suspended	Laboratory wet bench research suspended	All research suspended
**Trainee Management:**							
Have there been modifications to clinic or teaching for oncology residents?	None	Reduced outpatient oncology exposure	Reduced inpatient and outpatient exposure	None	Reduced outpatient oncology exposure	None	Reduced inpatient and outpatient exposure
Do you have off service residents completing oncology rotations?	Unchanged	No inpatient oncology exposure	No inpatient oncology exposure	No off service residents	Unchanged	Unchanged	No off service residents

Abbreviations: CT = computed tomography, MRI = magnetic resonance imaging, N/A = not applicable, PPE = personal protective equipment, CCI = Cross Cancer Institute, CACC = Central Alberta Cancer Centre, TBCC = Tom Baker Cancer Centre, JACC = Jack Ady Cancer Centre, G-CSF = granulocyte-colony stimulating factor, ACP = Advanced Care Planning, ICU = intensive care unit. Questionnaire completed: BC on June 23/20, AB on June 22/20, SK on June 26/20, MB on July 3/20, ON on Aug 25/20, QC on July 10/20, NL on July 4/20. Legend: ↓ = <25%, ↓↓ = 25–50%, ↓↓↓ = 50–75%, ↓↓↓↓ = >75%.

## Supplementary Materials

The following are available online at https://www.mdpi.com/1718-7729/28/1/26/s1, Table S1: Public provincial cancer centre and CAPCA electronic space information considering cancer screening, appointments, treatment and cancer centre operations changes.
