# Supplementary material for: Canadian Cancer Centre Response to COVID-19 Pandemic: A National and Provincial Response
Source: Curr Oncol. 2020 Dec 31;28(1):233–51. doi: 10.3390/curroncol28010026 (PMC7900889; doi:10.3390/curroncol28010026)
Supplement: Supplementary file 1 [file curroncol-28-00026-s001.zip › curroncol-1018633 suppl XML.pdf]

Table S1: Public provincial cancer centre and CAPCA electronic space information considering cancer screening, appointments, treatment and cancer centre operations changes

	British Columbia	Alberta	Saskatchewan	Manitoba	Ontario	Quebec	Nova Scotia	Newfoundland and Labrador
**Screening programs**	Breast, colorectal and cervical screening programs were temporarily suspended.<br><br>Some screening mammography will resume in June 2020. FIT is now available.	Breast, colorectal and cervical screening programs were temporarily suspended.	Breast, colorectal and cervical screening programs were temporarily suspended.<br><br>Some screening mammography will resume in June 2020. PAP smears and FIT testing are now available.	Breast, colorectal and cervical screening programs were temporarily suspended.<br><br>Some screening mammography will resume in June 2020. FOBT is now available.	Breast, colorectal and cervical screening programs were temporarily suspended.	Breast, colorectal and cervical screening programs were temporarily suspended.	Breast, colorectal and cervical screening programs were temporarily suspended.	-
**Outpatient appointments (new consults, on treatment, follow up)**	New consultations, on-treatment visits and follow up appointments may be completed in person or by virtual means. Generally, patients are to attend appointments alone.	New consultations, on-treatment visits and follow up appointments may be completed in person or by virtual means. Stable >6 month follow up appointments were deferred. Generally, patients are to attend appointments alone.	New consultations, on-treatment visits and follow up appointments may be completed in person or by virtual means. One support person is allowed to accompany a patient for the first appointment.	New consultations, on-treatment visits and follow up appointments may be completed in person or by virtual means. Generally, patients are to attend appointments alone.	New consultations, on-treatment visits and follow up appointments may be completed in person or by virtual means. Stable >6 month follow up appointments were deferred. Generally, patients are to attend appointments alone.	New consultations, on-treatment visits and follow up appointments may be completed in person or by virtual means. Generally, patients are to attend appointments alone.	New consultations, on-treatment visits and follow up appointments may be completed in person or by virtual means. One support person is allowed to accompany a patient for the first appointment.	Generally, patients are to attend new consultations, on-treatment visits and follow up appointments alone.
**Virtual platforms used (for patient appointments, case conference rounds)**	Virtual appointments include telephone and video capable platforms. Group meetings capable of meeting over video used platforms including: MOVI or Skype.	Virtual appointments include telephone and video capable platforms (over AHS Zoom).	Virtual appointments include telephone and video capable platforms.	Virtual appointments include telephone and video capable platforms (MicrosoftTeams).	Virtual appointments include telephone and video capable platforms.	Virtual appointments include telephone and video capable platforms.	Virtual appointments include telephone and video capable platforms (may include Zoom).	-
**Patient screening before entering Cancer Centre**	-	At entrance, patients are screened for symptoms and temperature measured.	At entrance, patients are screened for symptoms.	At entrance, patients are screened for symptoms.	At entrance, patients are screened for symptoms.	At entrance, patients are screened for symptoms	At entrance, patients are screened for symptoms	At entrance, patients are screened for symptoms
**Screening of health care professionals before Cancer Centre entrance**	-	To complete daily fit for work screening questionnaire within two hours of reporting for their shift.	-	At entrance, all cancer centre employees undergo screening questionnaire and have temperature measured.	At entrance, all cancer centre employees undergo screening questionnaire.	-	Self-monitor for symptoms.	-

	British Columbia	Alberta	Saskatchewan	Manitoba	Ontario	Quebec	Nova Scotia	Newfoundland and Labrador
**Imaging diagnostics**	Reduction in imaging and prioritization depending on indication. Phase 0-4 prioritization level for reduction in imaging depending on severity of pandemic.	Reduction in imaging and prioritization depending on indication.	-	Reduction in imaging and prioritization depending on indication.	Reduction in imaging and prioritization depending on indication. Consider deferral of interventional procedures (including port-a-catheter insertions, fine needle aspirations).	-	Reduction in imaging and prioritization depending on indication.	-
**Phlebotomy**	-	Phlebotomy for stable conditions should be postponed.	Phlebotomy for stable conditions should be postponed.	-	-	Phlebotomy should be completed close the patient's home and obtained 24-72 hours before treatment or appointment.	Launched home phlebotomy for patient's unable leave their home due to chronic illness, mobility concerns or in isolation due to COVID-19.	-
**Surgery**	Surgeries pertaining to cancer patients were to be some of the last surgeries delayed as the pandemic unfolded.	-	-	-	Surgeries were delayed based on priority level including cancer surgeries.	Surgeries were delayed based on priority level including cancer surgeries.	Surgeries pertaining to cancer patients may have experienced delays.	-
**Radiation Therapy**	Recommended to use the more efficient fractionation protocol. Minimize administration of radiation outside of normal business hours.	Recommended to use the more efficient fractionation protocol. Delay initiation of radiation if safe.	Radiation administration prioritization criteria were developed if resources were strained.	Recommended to use more efficient fractionation protocol. Prioritization criteria were developed if resources were strained.	If at all possible, patients who initiated radiation should complete course. If safe approaches exist to delay radiation initiation, they should be explored.	-	Recommended to use the more efficient fractionation protocol.	-
**Chemotherapy**	Longest possible chemotherapy cycle with the fewest number of appointments should be selected. Consider converting IV therapies to oral if equivalent.	Delay initiation of chemotherapy if safe.	-	If treatment is non-curative and patient's symptoms are well controlled, consider delaying treatment.	-	Consider risk versus benefit for chemotherapy if added benefit to chemotherapy is modest. Special attention should be taken for: patients >60 years of age or ECOG >2. Consider converting IV therapies to oral if equivalent. Use the longest possible cycle.	-	-
**Supportive Therapies**	Defer supportive therapies, such as adjuvant bisphosphonates. Consider adding G-CSF as primary prophylaxis.	-	-	-	-	-	Delay all adjuvant bisphosphonate therapy for breast cancer. Change monthly bisphosphonate therapy for metastatic disease to every 3 months.	-

Abbreviations: FIT = fecal immunochemical tests, PAP = papanicolaou, FOBT = fecal occult blood test, IV = intravenous, G-CSF = granulocyte-colony stimulating, ECOG = eastern cooperative oncology group
