# Supplementary material for: Canadian Cancer Centre Response to COVID-19 Pandemic: A National and Provincial Response
Source: Curr Oncol. 2020 Dec 31;28(1):233–51. doi: 10.3390/curroncol28010026 (PMC7900889; doi:10.3390/curroncol28010026)
Supplement: Supplementary file 1 [file curroncol-28-00026-s001.zip › Supplemental Appendix 1 - Questionnaire.v1.pdf]

Please mark your responses with an X to all that apply, and add comments as needed.

SCREENING PROGRAMS

Which provincial screening programs are still running?

None		Breast		Colorectal		Cervical		Lung		Other	

Which screening programs are you considering to re-start in the next 4 weeks?

None		Breast		Colorectal		Cervical		Lung		Other	

DIAGNOSTICS

What has been the impact to diagnostic services?

1. CT scan

None		Reduced by <25%		Reduced by 25-50%		Reduced by 50-75%		Reduced by >75%	

2. Ultrasound

None		Reduced by <25%		Reduced by 25-50%		Reduced by 50-75%		Reduced by >75%	

3. MRI

None		Reduced by <25%		Reduced by 25-50%		Reduced by 50-75%		Reduced by >75%	

4. Nuclear Medicine (Bonescan/MUGA/PET)

None		Reduced by <25%		Reduced by 25-50%		Reduced by 50-75%		Reduced by >75%	

5. Echocardiography

None		Reduced by <25%		Reduced by 25-50%		Reduced by 50-75%		Reduced by >75%	

6. CT/Ultrasound-guided Biopsy

None		Reduced by <25%		Reduced by 25-50%		Reduced by 50-75%		Reduced by >75%	

Has the reduction been for specific indications?

N/A		Staging		Follow-up		Other	

As you consider re-opening strategies, how long do you expect diagnostic restrictions to be in place?

N/A		<4 weeks		4-8 weeks		>8 weeks	

Have there been any changes in location that blood work is being completed?

No change		Shift to cancer centre		Shift away from cancer centre	

<u>OUTPATIENT APPOINTMENTS</u>

Has there been a decrease in new referrals to your centre?

No change		<10 decrease		10-20% decrease		>20% decrease	

Are you expecting a surge of new referrals once you re-open?

No change		<10 increase		10-20% increase		>20% increase	

Are you screening/testing patients prior to entering the cancer centre?

No		Screening questions and temperature		Nasopharyngeal swab prior to visit	

Have you asked patients to come alone?

No		Yes, requested		Yes, enforced	

Have you reduced the number of in person appointments allowed per clinic?

No change		<10 decrease		10-20% decrease		>20% decrease	

What percentage of outpatient appointments are occurring virtually (video or telephone)?

None		< 25%		25-50%		50-75%		> 75%	

i. New patient appointments:

None		< 25%		25-50%		50-75%		> 75%	

ii. On treatment appointments:

None		< 25%		25-50%		50-75%		> 75%	

iii. Follow up appointments:

None		< 25%		25-50%		50-75%		> 75%	

For virtual appointments, what percentage are delivered by video conferencing?

None		< 25%		25-50%		50-75%		> 75%	

For video virtual appointments, which platform is being used?

Microsoft Teams		Zoom		Pexip		Reacts		Other (specify)	

How are you communicating with patients about upcoming appointments?

Canada Post		Phone		Email		Patient Portal	

<u>INPATIENT WARDS / CONSULT SERVICE</u>

Have admissions to inpatient units decreased?

No change		Increase		<10% decrease		10-20% decrease		>20% decrease	

Has staffing of inpatient units changed?

No change		Fewer staff due to infection/quarantine		More staff due to inpatient volume		Staff redeployed to inpatient units	

Are patients being screened or tested for COVID-19 prior to admission?

No		Screening questions and temperature		Nasopharyngeal swab		Serologic testing	

How are you handling suspect or confirmed COVID-19 patients on your oncology wards?

Separate Unit		Negative Pressure rooms		Positive patients sharing rooms		Patients given N95	

TREATMENT

SURGERY

Have there been any delays or deferral of cancer surgeries?

None		< 4 weeks		4-6 weeks		6-8 weeks		> 8 weeks	

Have cancer surgeries been prioritized?

No change		Prioritization within disease sites		Certain disease sites prioritized higher (specify)		Prioritization of procedures to maximize surgical volume		All surgery delayed	

What will limit increases in surgical volume once you begin to re-open?

No change		Access to Personal Protective Equipment		COVID-19 precaution procedures (disinfection, PPE, locked room post intubation, etc)		Non-cancer surgery prioritization		Staffing	

RADIATION

Have there been any changes to the delivery of radiation therapy?

No change		Delayed where possible		Changes to dose or fractionation schedule		Prioritization within disease site/greatest benefit		Prioritization to specific disease sites (specify)		Prioritization to clinical indication (urgent, palliative, curative)	

How are you planning for increased volume once restrictions are eased?

No change		Increased LINAC hours		Continued use of modified dose schedule		Prioritization within disease site/greatest benefit		Prioritization to specific disease sites (specify)		Prioritization to clinical indication (urgent, palliative, curative)	

SYSTEMIC THERAPY

Have there been any changes to the administration of chemotherapy or supportive therapy?

No change		Delayed where possible		Changes to schedule (frequency, dose density, etc)		Prioritization within disease sites/greatest benefit		Prioritization to specific disease sites (specify)		Prioritization to clinical indication (urgent, palliative, curative)	

What additional systemic therapy considerations have been used?

No change		Favour oral therapy		Favour use of G-CSF		Favour neoadjuvant treatment		Favour pre-specified benefit threshold		Stopping maintenance therapy	

Have you had to adjust the drug approval process/policy to allow more flexibility in choosing regimens?

No Change		Drug access liberalized for pandemic		Access remains restricted		Drug funding remains a barrier		Disease site-specific liberalized access granted	

Have you modified the use of satellite chemotherapy sites?

No change		Increased use to reduce tertiary centre volume		<20% decrease		>20% decrease	

What changes have occurred in the treatment room?

No change		Distancing of patients		No escorts		Changed hours	

Are you testing patients prior to systemic therapy?

No		Certain regimens/disease sites		Yes, prior to cycle 1		Yes, prior to each cycle	

Will you be testing patients prior to systemic therapy?

No		Certain regimens/disease sites		Yes, prior to cycle 1		Yes, prior to each cycle	

Have you experienced any medication shortages?

No		Yes, oral systemic therapy		Yes, intravenous systemic therapy		Yes, supportive care	

ADVANCED CARE PLANNING (ACP)

Has there been direction to physicians to emphasize ACP or end of life care discussions in preparation for changes to access to ICU care?

No		Yes	

Have you used video appointments for psychosocial oncology care?

None		< 25%		25-50%		50-75%		> 75%	

Has the volume of psychosocial visits increased during this time?

No change		Decreased		< 20% increase		20-50% increase		> 50% increase	

CANCER CENTRE OPERATIONS

What platforms are being used for case conferences?

In person		Hybrid in person with distancing and video conference		Video conference	

Microsoft Teams		Zoom		Pexip		Reacts		Other (specify)	

Once restrictions ease, what services do you expect to continue long term?

Telephone visits		Video Visits		Electronic communication with patients		Video case conferences	

Are some staff members working from home?

No		<20%		20-50%		>50%		Variable/Scheduled rotation	

Physicians		Nursing		Health Records		Administration		Allied Health	

What is the biggest barrier to working from home?

None		Paper/in-person workflow		Lack of remote access		Lack of devices	

None		Paper/In-person workflow		Lack of remote access		Lack of devices		HR/union-related challenges	

Are patients or staff required to wear personal protective equipment (PPE)?

No		All staff (masks)		All patients (masks)		Direct patient care only	

What PPE are you using during a routine clinical encounter?

None		Eye protection		Surgical/Procedure mask		N95 mask		Gown		Gloves	

Have there been concerns relating to shortages of PPE?

No		Yes, staff limited to one PPE set per day		Yes, limiting number of providers seeing a patient		Yes, using what is available		Yes, limiting trainees to preserve PPE	

RESEARCH

Are clinical trials currently open at your cancer centre?

No, all activity suspended		Yes, but not open to recruitment		Yes, limited trials open		Yes, all trials open		Yes, phased in plan to open trials	

Has non-clinical trial research continued?

Yes, no restrictions		All research suspended		Clinical research suspended		Laboratory wet bench research suspended		Database/in silico/health services research suspended	

TRAINEE MANAGEMENT

Have there been modifications to clinic or teaching for oncology residents?

None		Shifted to support inpatient care		Reduced inpatient oncology exposure		Reduced outpatient oncology exposure		Rotations completely suspended	

Do you have off service residents completing oncology rotations?

No		Shifted to support inpatient care		No inpatient oncology exposure		No outpatient oncology exposure		Unchanged	
